# Estrous Cycle-Dependent Expression of Nucleobindin-2/Nesfatin-1 in the Porcine Hypothalamus: Colocalization with GnRH and Kisspeptin

**DOI:** 10.3390/cells15211919

**Published:** 2026-10-22

**Authors:** Milena Traut, Ewa Zaobidna, Aleksandra Tkacz, Kamil Dobrzyn, Marta Kiezun, Barbara Kierczuk, Marlena Gudelska, Katarzyna Kisielewska, Agnieszka Rak, Nina Smolinska, Tadeusz Kaminski

**Affiliations:** 1 Department of Animal Anatomy and Physiology, Faculty of Biology and Biotechnology, University of Warmia and Mazury in Olsztyn, Oczapowskiego 1A, 10-719 Olsztyn, Poland; milena.traut@uwm.edu.pl (M.T.); aleksandra.tkacz@doktorant.uwm.edu.pl (A.T.); kamil.dobrzyn@uwm.edu.pl (K.D.); marta.kiezun@uwm.edu.pl (M.K.); barbara.zarzecka@doktorant.uwm.edu.pl (B.K.); nina.smolinska@uwm.edu.pl (N.S.); 2 Department of Biochemistry, Faculty of Biology and Biotechnology, University of Warmia and Mazury in Olsztyn, Oczapowskiego 1A, 10-719 Olsztyn, Poland; ewa.zaobidna@uwm.edu.pl; 3 Department of Anatomy and Histology, School of Medicine, Collegium Medicum, University of Warmia and Mazury in Olsztyn, Warszawska 30, 10-082 Olsztyn, Poland; marlena.gudelska@uwm.edu.pl (M.G.); katarzyna.kisielewska@uwm.edu.pl (K.K.); 4 Laboratory of Physiology and Toxicology of Reproduction, Institute of Zoology and Biomedical Research, Jagiellonian University in Krakow, Gronostajowa 9, 30-387 Krakow, Poland; agnieszka.rak@uj.edu.pl

**Keywords:** NUCB2, nesfatin-1, hypothalamus, pig, estrous cycle

## Abstract

Nesfatin-1, an 82-amino-acid anorexigenic peptide derived from nucleobindin-2 (NUCB2), is involved in the regulation of energy homeostasis and is increasingly recognized as a modulator of reproductive function. The present study aimed to determine *NUCB2* gene and protein expression in the porcine hypothalamus, examine their colocalization with gonadotropin-releasing hormone (GnRH) and kisspeptin (KISS1), and evaluate circulating nesfatin-1 levels during the estrous cycle. Analyses were performed in two hypothalamic regions: the mediobasal hypothalamus (MBH) and preoptic area (POA) collected during the cycle. In the MBH, *NUCB2* gene expression was lower on days 17–19 than on the remaining examined days, whereas NUCB2 protein abundance was highest on days 2–3. In the POA, *NUCB2* mRNA expression peaked on days 14–16, while the protein abundance was higher on days 10–12 than on days 14–16. In both hypothalamic regions, NUCB2/nesfatin-1 immunoreactivity overlapped with GnRH and KISS1 immunosignals. In addition, plasma nesfatin-1 concentrations were lower on days 17–19 than during the other examined stages of the cycle. Overall, these findings demonstrate cycle-dependent, region-specific expression of NUCB2/nesfatin-1 in the porcine hypothalamus and support a potential association of nesfatin-1 with hypothalamic pathways involved in reproductive regulation.

## 1. Introduction

Nesfatin-1 is an 82-amino-acid anorexigenic peptide involved in the regulation of energy homeostasis identified in 2006 [1]. Its precursor, nucleobindin 2 (NUCB2), consists of a 24-amino-acid signal peptide and a 396-amino-acid polypeptide. Post-translational processing of NUCB2 by prohormone convertases yields three fragments: nesfatin-1 (amino acids 1–82), nesfatin-2 (85–163), and nesfatin-3 (166–396) [2]. To date, no biological activity has been attributed to nesfatin-2 or nesfatin-3. Although the specific receptor for nesfatin-1 remains unidentified, binding sites for ^125^I-nesfatin-1 have been reported in the hypothalamus, pituitary gland, and testis [3]. The biological effects of nesfatin-1 appear to be mediated through activation of several signaling pathways, including adenylate cyclase/protein kinase A (AC/PKA), AMP-activated protein kinase (AMPK), phosphoinositide 3-kinase/protein kinase B (PI3K/Akt), and mitogen-activated protein kinase/extracellular signal-regulated kinase (MAPK/ERK1/2) pathways [4,5,6]. Given its role in energy homeostasis and its emerging involvement in reproductive function, nesfatin-1 may act as an integrative endocrine signal between metabolic, stress-related, and reproductive pathways across the reproductive life course [7]. NUCB2/nesfatin-1 is a peptide widely distributed in both the central nervous system and peripheral tissues [8]. It has been detected in the hypothalamus of pigs [9], mice [10], rats [11], and goldfish [12], as well as in the hypothalamic GT1-7 cell line [13]. In rats, hypothalamic *NUCB2* gene expression varies with metabolic status; fasting reduces both *NUCB2* expression and nesfatin-1 levels in hypothalamic nuclei, indicating sensitivity to energy balance [1]. NUCB2/nesfatin-1 has also been identified in the pituitary gland [14] and in the pituitary LβT2 cell line [13]. Beyond the central nervous system, NUCB2/nesfatin-1 has been detected in both male and female reproductive tissues [11]. In mice, it is present in the uterus and ovaries [10,15], whereas in pigs it has been identified in ovarian follicles [16,17].

In rodents, NUCB2/nesfatin-1 expression within the hypothalamic–pituitary–gonadal (HPG) axis is influenced by sexual maturation, the estrous cycle, gonadectomy, and the actions of estradiol (E_2_), progesterone (P_4_), testosterone (T), and gonadotropins [10,11,14,18,19,20], indicating tight hormonal regulation. In female rats, circulating nesfatin-1 levels correlate positively with follicle-stimulating hormone (FSH), E_2_, and P_4_, and negatively with luteinizing hormone (LH) and T [21]. The localization of nesfatin-1 within HPG axis structures suggests its involvement in reproductive function. Indeed, nesfatin-1 appears to influence maturation and reproductive development in rodents. In rats, intracerebroventricular (i.c.v.) infusion of an anti-NUCB2 antisense oligonucleotide during puberty delayed vaginal opening and reduced ovarian weight and serum LH levels [18]. In contrast, in male rats, central administration of nesfatin-1 decreased hypothalamic GnRH and kisspeptin mRNA expression, as well as pituitary FSHβ and LHβ transcripts, leading to reduced serum FSH and LH concentrations [11]. Comparable inhibitory findings were reported in fish, where nesfatin-1 acutely suppressed hypothalamic GnRH transcripts, pituitary gonadotropin expression, circulating LH, and oocyte maturation [12,22], whereas in pigs it promotes cleavage and blastocyst development [17]. These findings suggest that nesfatin-1 may act in a species-diverse manner at the hypothalamic level to convey information about energy availability to GnRH and kisspeptin neurons, thereby influencing the HPG axis. However, such studies remain scarce in farm animals, including pigs. Based on the above data, we hypothesize that nesfatin-1 is expressed in porcine hypothalamic regions involved in the control of GnRH production, and that both its expression and plasma concentration vary across the estrous cycle. Therefore, the aim of the present study was to determine *NUCB2* gene expression and NUCB2 protein abundance in two porcine hypothalamic structures, the mediobasal hypothalamus (MBH) and the preoptic area (POA), to examine the localization and colocalization of NUCB2/nesfatin-1 with GnRH and kisspeptin (KISS1), and to assess circulating nesfatin-1 levels during key stages of the estrous cycle.

## 2. Materials and Methods

### 2.1. Materials

#### 2.1.1. Animals

Animals were selected as previously described [23]. All procedures were conducted in accordance with the Polish Act of 15th January 2015 on the protection of animals used for scientific or educational purposes (Journal of Laws of the Republic of Poland 2015, No. 266) and Directive 2010/63/EU of the European Parliament and of the Council of the 22nd of September 2010 on the protection of animals used for scientific purposes. The study included mature crossbred gilts aged 7–8 months with an average body weight of 140–150 kg. The animals were fed a balanced diet that met Polish nutritional requirements for pigs and had free access to fresh water. Tissue samples were collected exclusively from animals intended for commercial slaughter and subsequent meat processing. To evaluate hypothalamic expression of nesfatin-1, a total of twenty animals were assigned to four experimental groups (*n* = 5 per group). The experimental groups represented four phases of the estrous cycle: days 2–3 (early-luteal phase; presence of corpora haemorrhagica), days 10–12 (mid-luteal phase; period of maximal corpus luteum activity), days 14–16 (late-luteal phase; luteolysis) and days 17–19 (follicular phase). The stage of the estrous cycle was determined based on ovarian morphology as described by Akins and Morisette [24].

#### 2.1.2. Blood Samples and Tissue Collection

After slaughter, blood samples were collected from the jugular vein into heparinized tubes, transported to the laboratory on ice, and centrifuged at 2500× *g* for 15 min at 4 °C to obtain plasma. Plasma samples were stored at −20 °C until analysis. The hypothalamic block was excised from the brain and dissected into two regions: the MBH and POA, as previously described [25]. The MBH was defined as the region located posterior to the optic chiasma, anterior to the mammillary body, laterally bordered by the hypothalamic sulci, and dorsally limited by a 5 mm deep incision. The POA was defined as the region located approximately 5 mm rostral to the optic chiasma. MBH and POA samples collected on days 10–12 of the estrous cycle were divided into two equal parts: one half was fixed in 4% buffered paraformaldehyde (pH = 7.4, 4 °C) for immunofluorescent staining (F-IHC), whereas the other half was frozen in liquid nitrogen and stored at −80 °C for RNA and protein analyses.

#### 2.1.3. Fluorescent Immunohistochemistry

Fluorescent immunohistochemistry was performed on 8-μm paraffin-embedded sections of the MBH and POA (*n* = 4) to assess localization and colocalization of NUCB2/nesfatin-1 with GnRH or KISS1. To retrieve antigen epitopes, sections were incubated with 0.25% trypsin (cat. no. TRY-3B; Biomed Lublin, Poland) at 37 °C for 30 min. To reduce nonspecific background fluorescence, sections were incubated for 30 min with 50 mM NH_4_CL in 0.01 M phosphate-buffered saline (PBS; pH 7.4), permeabilized in 0.1% Triton™ X-100, and then treated with Image-iT™ FX Signal Enhancer (cat. no. R37107; Thermo Fisher Scientific, Waltham, MA, USA) for 30 min. Nonspecific binding sites were blocked for 1.5 h at room temperature using Fish Serum Blocking Buffer (cat. no. 37527; Thermo Fisher Scientific, Rockford, IL, USA). Sections were then incubated overnight at 4 °C with mouse monoclonal anti-NUCB2/nesfatin-1 antibody raised against nesfatin-1 and reacting both with full-length NUCB2 and nesfatin-1 (1:100; cat. no. MAbs1164; Merck, Darmstadt, Germany), combined with mouse monoclonal anti-GnRH antibodies (1:100; cat. no. A304433; antibodies.com, Cambridge, UK) or rabbit polyclonal anti-KISS1 antibodies (1:100; cat. no. 18375-1-AP; Proteintech, Rosemont, IL, USA). After washing, the sections were incubated for 1.5 h at room temperature with Alexa Fluor™ 488-conjugated goat anti-mouse secondary antibodies (1:1000; cat. no. A32723TR; Thermo Fisher Scientific, Waltham, MA, USA) and Alexa Fluor™ 594-conjugated goat anti-rabbit secondary antibodies (1:1000; cat. no. A32742; Thermo Fisher Scientific, Waltham, MA, USA). Negative control sections were processed in parallel by omitting the primary antibodies. Tissue autofluorescence was reduced using the TrueVIEW™ Autofluorescence Quenching Kit (cat. no. SP-8400; Vector Laboratories, Newark, CA, USA). The sections were air-dried and mounted in Fluoroshield™ medium (cat. no. F6057; Merck, St. Louis, MO, USA) with DAPI for nuclear counterstaining. The labelled sections were analyzed using an Olympus BX51 fluorescence microscope (Olympus, Tokyo, Japan) equipped with an EXFO X-Cite™ series 120Q fluorescence illuminator (Excelitas Technologies Corp., Pittsburgh, PA, USA) and appropriate filter sets for DAPI and Alexa Fluor^®^ fluorophores. Images were acquired using a Nikon DS-Qi2 digital camera (Nikon, Tokyo, Japan) and cellSens Dimension 1.7 image-processing software (Olympus Soft Imaging Solutions, Münster, Germany).

#### 2.1.4. Quantitative Real-Time PCR (qPCR)

Total RNA was extracted using TRI reagent (Sigma-Aldrich, St. Louis, MO, USA) according to the manufacturer’s instructions, as previously reported [23]. RNA concentration and quality were assessed spectrophotometrically using an Infinite M200 Pro reader (Tecan, Männedorf, Switzerland). RNA extraction, cDNA synthesis, and qPCR procedures were performed as previously outlined [26]. QPCR was conducted using an AriaMx Real-Time PCR System (Agilent Technologies, Santa Clara, CA, USA) and Sensitive RT HS-PCR Mix SYBR^®^ (cat. no. 2008HS-100, A&A Biotechnology, Gdańsk, Poland). Specific primers for *NUCB2*, *UBC* (ubiquitin C), and *18sRNA* (18S ribosomal RNA) used in the study are listed in Table 1. *UBC* and *18sRNA* served as reference genes because their Ct values remained stable across both tissues and all phases of the estrous cycle. The qPCR reaction mixtures and thermal parameters are outlined in Table 1. Each reaction was performed in duplicate in a final volume containing 20 ng of cDNA, appropriate concentrations of forward and reverse primers (Table 1), 12.5 µL of RT-PCR Mix SYBR^®^ (cat. no. 1032-100, A&A Biotechnology, Gdansk, Poland), and RNase-free water. Amplification specificity was verified by melting-curve analysis. Relative gene expression was calculated using the comparative cycle threshold method (2^−∆∆Ct^) and normalized to the geometric mean of *UBC* and *18sRNA* expression. No amplification was detected in non-template controls.

#### 2.1.5. Western Blot

Western blot analyses were performed as previously described [29] with minor modifications. Briefly, 30 mg samples of the porcine MBH and POA tissue were homogenized in 500 µL of T-PER Tissue Protein Extraction Reagent (cat. no. 78510; Thermo Fisher Scientific, Waltham, MA, USA). Equal amounts of protein (40 μg protein per sample) were separated by SDS-PAGE on 12.5% gels and transferred onto 0.2 µm PVDF membranes. Membranes were blocked for 1 h at room temperature in 0.02 M TBS containing 0.1% Tween 20 (TBST) and 5% bovine serum albumin (BSA), and then incubated overnight at 4 °C with primary rabbit polyclonal anti-NUCB2/nesfatin-1 antibodies (1:100; cat. no. QD161202; Enlibio Biotech Co., Ltd., Wuhan, Hubei, China) and anti-actin antibodies (1:5000; cat. no. A2066; Sigma-Aldrich, St. Louis, MO, USA). Similarly, as in the case of immunohistochemical studies, the anti-NUCB2/nesfatin-1 antibody used in the present study was selected because commercially available antibodies with validated specificity for mature nesfatin-1 (~9.5 kDa) suitable for conventional Western blotting were unavailable. Actin immunoreactivity was used as the loading control. After washing in TBST, membranes were incubated for 1 h at room temperature with horseradish peroxidase-conjugated goat anti-rabbit secondary antibodies (1:5000; cat. no. #111-035-003; Jackson ImmunoResearch, West Grove, PA, USA). Signals were detected using Immobilon Western Chemiluminescent HRP Substrate (Merck Millipore, USA) and visualized with an Azure 280 imaging system (Azure Biosystems, Dublin, CA, USA). Densitometric analysis was performed using Image Studio™ Lite software (version 5.2; LI-COR, Lincoln, NE, USA). The abundance of the NUCB2/nesfatin-1-immunoreactive band was normalized to actin and expressed in arbitrary units.

#### 2.1.6. Enzyme-Linked Immunosorbent Assay (ELISA)

Plasma nesfatin-1 concentrations were measured using a commercial Porcine Nesfatin-1 ELISA Kit (cat. no. EIA08424p; Wuhan Enlibio Biotech, China) according to the manufacturer’s instructions. The assay sensitivity (minimum detectable concentration) was 0.05 ng/mL. The standard curve ranged from 0 to 10 ng/mL. Absorbance was measured at 450 nm using an Infinite M200 Pro microplate reader along with Tecan i-control software ver. 1.11 (Tecan, Männedorf, Switzerland). Nesfatin-1 concentrations were calculated by plotting the logarithm of nesfatin-1 concentration against the logarithm of the optical density, and the best-fit line was determined by regression analysis. The intra-assay coefficient of variation was 3.13%.

#### 2.1.7. Statistical Analysis

Data from qPCR, Western blot, and ELISA analyses were derived from five independent biological replicates, whereas fluorescent immunohistochemistry was performed on four biological replicates. Differences among groups were analyzed by one-way analysis of variance (ANOVA) followed by Tukey’s honestly significant difference post hoc test. The assumptions of normality and homogeneity of variances were assessed using the Shapiro–Wilk and Levene tests, respectively. Statistical analyses were performed using Statistica software (ver. 13.3 software, StatSoft Inc., Tulsa, OK, USA). Differences were considered statistically significant at *p* < 0.05.

## 3. Results

### 3.1. Localization and Colocalization of NUCB2/Nesfatin-1 with GnRH or KISS1 in the Porcine Hypothalamus

**Figure 1:**
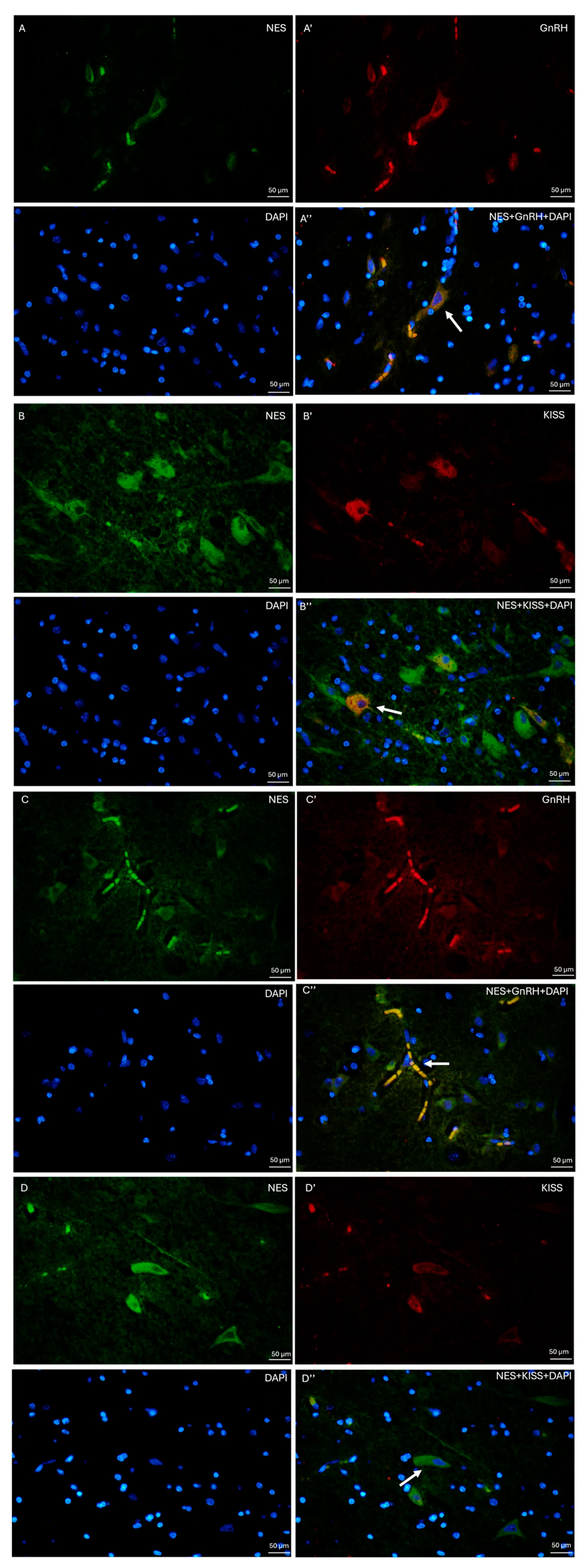
Immunofluorescence localization of NUCB2/nesfatin-1 and overlap with GnRH or KISS1 in the mediobasal hypothalamus (MBH) and the preoptic area (POA) collected on days 10–12 of the estrous cycle. In the MBH, immunofluorescence images show nucleobindin-2 (NUCB2)/nesfatin-1 (NES; **A**,**B**), DAPI nuclear staining (DAPI), and GnRH (gonadotropin-releasing hormone) (**A′**) or KISS1 (kisspeptin) (**B′**), together with merged images showing overlap of NUCB2/nesfatin-1 with GnRH (**A″**) or KISS1 (**B″**). In the POA, immunofluorescence images show NUCB2/nesfatin-1 (NES; **C**,**D**), DAPI nuclear staining (DAPI), and GnRH (**C′**) or KISS1 (**D′**), together with merged images showing overlap of NUCB2/nesfatin-1 with GnRH (**C″**) or KISS1 (**D″**). Magnification of 40×; scale bar 50 µm.

Immunofluorescent analysis revealed NUCB2/nesfatin-1-immunoreactive cells in both analyzed hypothalamic regions, the MBH and POA (Figure 1). No specific immunofluorescent signal was detected in the negative control sections. Double-label immunofluorescence revealed colocalization of NUCB2/nesfatin-1 immunoreactivity with GnRH- or KISS1-immunoreactive cells in both hypothalamic regions (Figure 1).

### 3.2. NUCB2 Gene Expression and NUCB2-Immunoreactive Protein Abundance in the Mediobasal Hypothalamus

**Figure 2:**
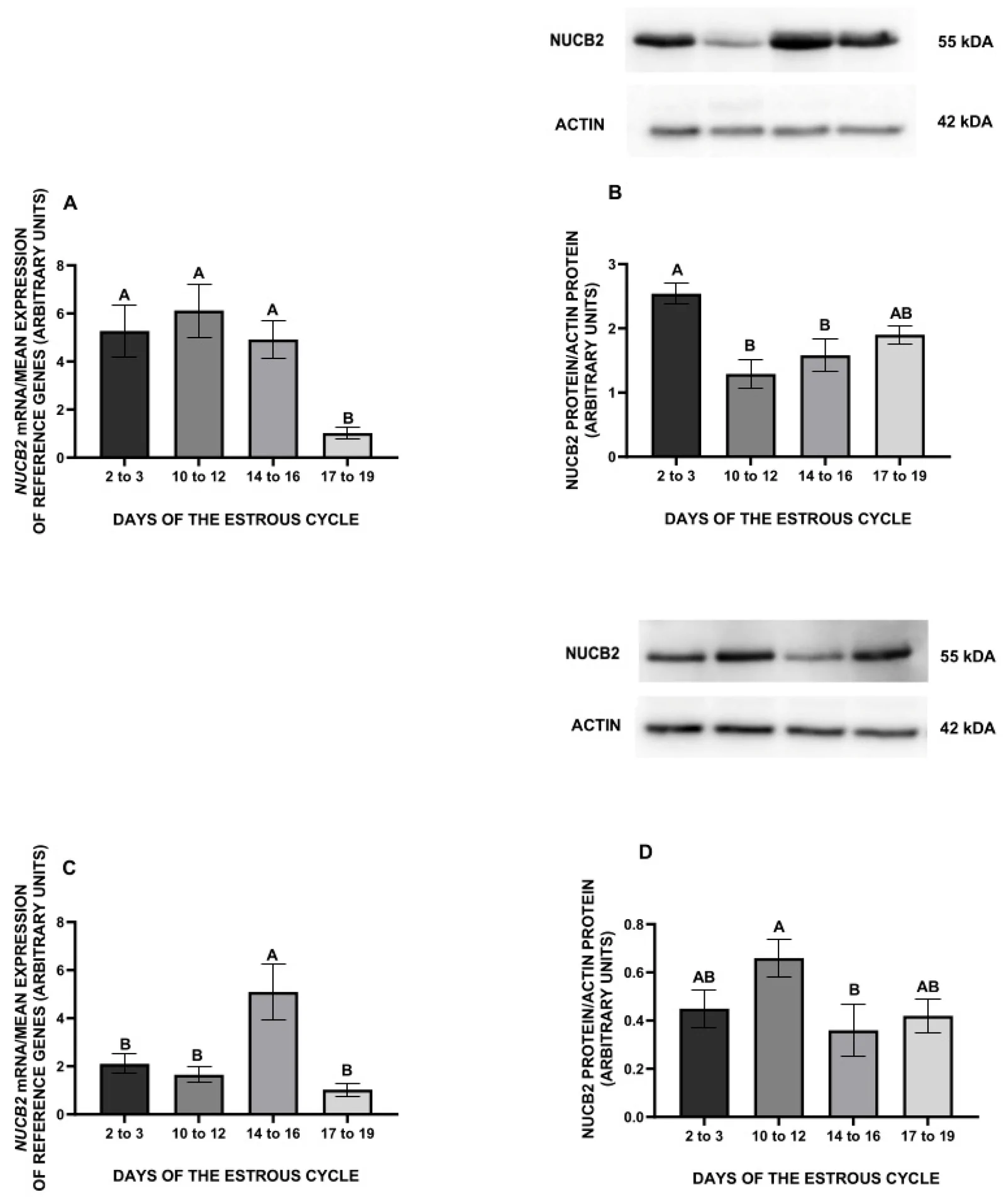
*NUCB2* gene expression and NUCB2 protein abundance in the porcine mediobasal hypothalamus (MBH) and preoptic area (POA) during the estrous cycle. The mRNA expression and the protein abundance were analyzed on days 2–3, 10–12, 14–16, and 17–19 of the estrous cycle by quantitative real-time PCR (qPCR) and Western blot, respectively. Panel (**A**) shows nucleobindin-2 (NUCB2) gene expression, and panel (**B**) shows NUCB2 protein levels in the MBH, whereas panels (**C**) and (**D**) show the corresponding data in the POA. For Western blot analysis, the upper panels show representative immunoblots, and the lower panels show densitometric analysis of NUCB2 protein normalized to actin (all bands shown in the figure come from the same blot). A single immunoreactive band of approximately 55 kDa was detected with the anti-NUCB2/nesfatin-1 antibody (1:100; cat. no. QD161202; Enlibio Biotech Co., Ltd., China). No immunoreactive band corresponding to the expected molecular weight of mature nesfatin-1 (~9.5–10 kDa) was detected in the MBH and POA. Data are presented as means ± SEM (*n* = 5). Bars with different superscript letters are significantly different (one-way ANOVA, *p* < 0.05).

In the MBH, *NUCB2* mRNA content was significantly lower during the follicular phase than during the early-, mid-, and late-luteal phases of the estrous cycle (*p* < 0.05; Figure 2A).

The abundance of the NUCB2 protein was highest during the early-luteal phase and was lower during the mid- and late-luteal phases (*p* < 0.05) of the cycle (Figure 2B). No significant differences were detected between the follicular phase and the other examined stages.

### 3.3. NUCB2 Gene Expression and NUCB2-Immunoreactive Protein Abundance in the Preoptic Area

In the POA, *NUCB2* gene expression was significantly higher during the late-luteal phase than during the other studied phases of the estrous cycle (*p* < 0.05; Figure 2C).

NUCB2 protein abundance in the POA was higher during the mid-luteal phase than during the late-luteal phase (*p* < 0.05; Figure 2D). No significant differences were detected between the early-luteal phase or follicular phase and the remaining stages of the cycle.

### 3.4. Plasma Nesfatin-1 Concentration

**Figure 3:**
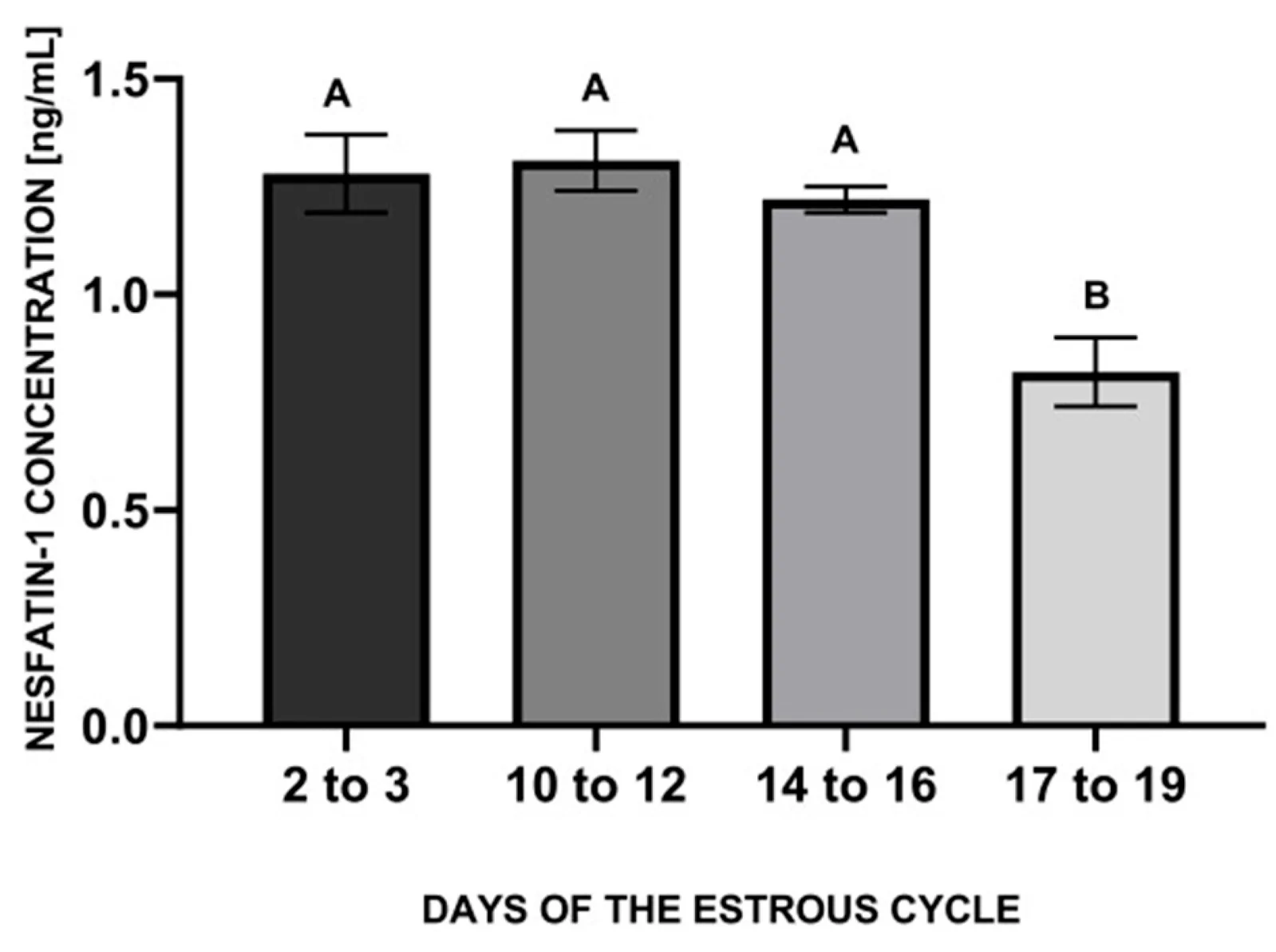
Plasma nesfatin-1 concentration in gilts during the estrous cycle, determined on days 2–3, 10–12, 14–16, and 17–19 by enzyme-linked immunosorbent assay (ELISA). Data are presented as mean ± SEM (*n* = 5). Bars with different superscript letters are significantly different (one-way ANOVA, *p* < 0.05).

Plasma nesfatin-1 concentration was lower in the follicular phase than in the early-, mid-, and late-luteal phases of the estrous cycle (*p* < 0.05; Figure 3).

## 4. Discussion

To the best of our knowledge, this is the first study to demonstrate circulating nesfatin-1 levels, hypothalamic *NUCB2* gene and NUCB2 protein expression, and localization together with GnRH and KISS1 in the porcine MBH and POA, which are key components of the neuroendocrine network regulating reproductive function [9]. The discrepancy between *NUCB2* gene expression and NUCB2 protein abundance observed in some cycle stages may reflect post-transcriptional and post-translational regulation, a phenomenon reported for many hypothalamic genes [23,30]. These differences could be attributed to several reasons, including transcriptional and post-transcriptional regulation (RNA processing and stability), translational regulation (lower rate of mRNA transcription compared to protein translation in mammalian cells), post-translational processes, the stability of mRNA and proteins, or physiological factors that could have an impact on the results obtained in the study [31,32]. Gry et al. [31] found that the correlation between gene and protein levels was not stronger than 40%. Therefore, in most cases, changes in mRNA and protein levels are not correlated, mainly because of regulatory mechanisms at different levels.

The hypothalamus appears to be a place of nesfatin-1 expression, as both *NUCB2* mRNA and protein were detected in this region. Our results indicate that *NUCB2* mRNA expression and NUCB2 protein abundance vary across the estrous cycle, which is consistent with observations in rodents. In rats, hypothalamic *NUCB2* mRNA content is reduced during proestrus compared with diestrus [33], whereas NUCB2/nesfatin-1 immunoreactivity in the paraventricular nucleus is increased during estrus and decreased during proestrus [20]. Cycle-dependent regulation has also been described in other components of the HPG axis, including the pituitary and ovaries. In the anterior pituitary of female rats, NUCB2/nesfatin-1 immunoreactivity decreases during proestrus compared with estrus and diestrus [20], and in the mouse ovaries, *NUCB2* mRNA and NUCB2 protein levels are elevated during estrus [10]. Together, these observations support the view that hypothalamic NUCB2 expression is dynamically regulated across the estrous cycle and may therefore be linked to the cyclic modulation of reproductive function.

The cycle-dependent changes observed in the present study may be related to the steroid milieu. Experimental studies in rodents show that sex steroids can modulate NUCB2/nesfatin-1 expression, although the direction and magnitude of this response appear to depend on tissue type, species, and experimental context. For example, T increased *NUCB2* mRNA and protein levels in the murine GT1-7 hypothalamic cell line [13], whereas incubation of male mouse hypothalamic tissue with T reduced *NUCB2* gene expression [19]. In the mouse pituitary, however, T appears to exert the opposite effect, as castration reduced *NUCB2* mRNA levels, whereas T replacement restored and further increased its expression [19]. Similarly, ovariectomy markedly decreased pituitary *NUCB2* mRNA content in mice, while administration of P_4_ and E_2_ increased transcript levels. Importantly, steroid hormones in the brain are not derived exclusively from the gonads but can also be synthesized locally, primarily by astrocytes [34]. Consequently, hypothalamic steroid levels depend on circulating concentrations, local metabolism, and de novo synthesis [35,36,37]. This process is further modulated by peripheral steroids, as demonstrated by the stimulatory effect of E_2_ on the expression and activity of 3β- hydroxysteroid dehydrogenase [38], a key enzyme in P_4_ synthesis. These findings indicate that steroid hormones may contribute to the regulation of the nesfatin-1 system; however, the specific mechanism appears to be tissue- and hormone-dependent.

Another notable finding is that the MBH and POA displayed distinct NUCB2 expression profiles across the estrous cycle. This may result from differences in the expression and density of estrogen and progesterone receptors across specific hypothalamic regions [39,40,41], leading to divergent neuronal responses to steroid hormones. A substantial body of evidence indicates that, in rodents, hypothalamic expression of these receptors varies across the estrous cycle and is regulated by E_2_ and P_4_ in a region-specific manner. Notably, the effects of E_2_ on the expression of estrogen receptors α and progesterone receptors have been demonstrated in hypothalamic KISS1 neurons [for review, see [42,43]]. Changes in estrogen receptor β mRNA expression during the estrous cycle have also been reported in the porcine MBH, with increased expression observed on days 9–14 compared with day 21 of the cycle [44]. Collectively, these findings suggest that the differential expression of NUCB2 in the MBH and POA observed in the present study may be driven by region-specific actions of sex steroid hormones.

Plasma nesfatin-1 concentrations remained relatively stable during the luteal phase and decreased on days 17–19, corresponding to the follicular phase. This pattern is consistent with the study of Ak et al. [20], who reported lower nesfatin-1 levels during proestrus in female rats. In another rat model, circulating nesfatin-1 correlated positively with FSH, E_2_, and P_4_, and negatively with LH and T [21], whereas no such relationship was found in girls with central precocious puberty [45]. These observations suggest that circulating nesfatin-1 may be influenced by the endocrine status of the animal, although additional metabolic and tissue-specific factors are also likely to contribute.

Immunofluorescence analysis revealed NUCB2/nesfatin-1 immunoreactivity in both examined hypothalamic regions and revealed overlapping signals with GnRH and KISS1. It is important to mention that the immunofluorescence analysis was performed only on tissues from the mid-luteal phase of the cycle. Although nesfatin-1 has been extensively studied in peripheral tissues, its distribution and potential neuroendocrine associations within the brain remain incompletely characterized. NUCB2/nesfatin-1 has been identified in several hypothalamic nuclei, including the arcuate, paraventricular, and supraoptic nuclei in rodents [11,13] and pigs [9]. These nuclei participate in pathways related to the control of GnRH and KISS1 signaling. Previous studies also demonstrated NUCB2/nesfatin-1/GnRH colocalization in GT1-7 cells and murine hypothalamic neurons [13]. Moreover, functional experiments on GT1-7 cells showed that nesfatin-1 increased KISS1 receptor gene expression as well as *GnRH* mRNA and protein levels, whereas KISS1 increased *NUCB2* mRNA and protein levels [13]. Nesfatin-1 was also shown to upregulate estrogen receptor mRNA expression in GT1-7 cells, suggesting that it may modulate the sensitivity of hypothalamic neurons to estrogen signaling [13].

In adult female rats, i.c.v. administration of nesfatin-1 did not affect FSH or LH secretion, whereas in pubertal females it increased serum gonadotropin concentrations [18]. By contrast, in male rats, i.c.v. administration of nesfatin-1 reduced pituitary FSHβ and LHβ expression as well as circulating FSH and LH levels [11], although another study in male rats reported increased plasma LH and FSH concentrations after similar treatment [46]. In prepubertal gilts, central administration of nesfatin-1 stimulated LH secretion [47]. Taken together, these findings indicate that the neuroendocrine actions of nesfatin-1 are context-dependent and may vary according to species, sex, developmental status, and experimental model. In pigs and, more broadly, in domestic animals, the role of nesfatin-1 in the regulation of hypothalamic and pituitary secretory activity remains insufficiently understood. The present findings provide anatomical and expression-based support for further studies addressing the functional role of nesfatin-1 in porcine reproductive neuroendocrinology.

Unfortunately, a significant methodological limitation of the study is the inability to unequivocally distinguish the mature form of nesfatin-1 from full-length nucleobindin-2 (NUCB2). In the present study, we did not detect the expected 9.5–10 kDa band for nesfatin-1 in the porcine hypothalamus. Although mature nesfatin-1 has a predicted molecular mass of approximately 9.7–10 kDa, commercially available anti-NUCB2/nesfatin-1 antibodies do not necessarily allow unequivocal discrimination between the precursor protein and its processed peptide. In the present study, an anti-NUCB2/nesfatin-1 antibody detected a prominent immunoreactive band at approximately 55 kDa, consistent with full-length NUCB2. This finding is in agreement with previous studies demonstrating that commercially available anti-nesfatin-1 antibodies can recognize full-length NUCB2, while endogenous mature nesfatin-1 is not reliably detected by conventional Western blotting [48,49,50,51]. To date, none of these studies has demonstrated endogenous, fully processed nesfatin-1 by Western blotting in any species, raising the possibility that its tissue levels are below the detection limit of this technique. It has also been proposed that NUCB2 may undergo processing predominantly upon secretion into the circulation, generating the ~9.5 kDa nesfatin-1 peptide [49]. Therefore, the absence of the ~10 kDa band cannot be considered definitive evidence for the absence of nesfatin-1, whereas the ~55 kDa band should primarily be interpreted as full-length NUCB2. Another limitation is that we did not determine whether the observed cycle-dependent changes in NUCB2 are associated with changes in GnRH secretion. Thus, our results indicate cycle-dependent regulation of the NUCB2/nesfatin-1 system but do not provide direct evidence for its involvement in GnRH pulsatility or the preovulatory stage. Further studies directly assessing GnRH dynamics are needed to clarify this association.

## 5. Conclusions

In conclusion, the present study demonstrates that the porcine hypothalamus expresses NUCB2 in a region- and cycle-dependent manner. The overlap of NUCB2/nesfatin-1 immunosignals with GnRH and KISS1 in the MBH and POA, together with changes in plasma nesfatin-1 concentrations across the estrous cycle, supports a potential association of this peptide with hypothalamic pathways involved in reproductive regulation. These findings extend previous observations from rodent models to pigs and support a potential role for the NUCB2/nesfatin-1 system in regulating reproductive function. However, the present study does not establish a direct functional effect of nesfatin-1 on GnRH or kisspeptin signaling. Further mechanistic studies are needed to clarify how nesfatin-1 interacts with the porcine HPG axis.

## Figures and Tables

**Table 1:** Characteristics of primers used in the study.

Gene Symbol	Primer Sequences	Reaction Conditions	Number of Cycles	Primer (nM)	Target Sequence Accession Number	References
*NUCB2*	F: 5′-TGCGAAGATAGAACCACCAG-3′ R: 5′-GCCACTTCTTGCCTTTTCAG-3′	Activation: 95 °C, 3 min Denaturation: 95 °C, 30 s Annealing: 60 °C, 1 min Elongation: 70 °C, 30 s	40	200	XM_021083276.1	[27]
*UBC*	F: 5′-GGAGGAATCTACTGGGGCGG-3′ R: 5′-CAGAAGAAACGCAGGCAAACT-3′	Activation: 95 °C, 3 min Denaturation: 95 °C, 30 s Annealing: 60 °C, 1 min Elongation: 70 °C, 30 s	40	400	XM_003483411.3	[28]
*18sRNA*	F: 5′-TCCAATGGATCCTCGCGG AA-3′ R: 5′-GGCTACCACATCCAAGGAAG-3′	Activation: 95 °C, 3 min Denaturation: 95 °C, 30 s Annealing: 60 °C, 1 min Elongation: 70 °C, 30 s	40	400	AY265350.1	[28]

*NUCB2*, nucleobindin-2; *UBC*, ubiquitin C; *18sRNA*, 18s ribosomal RNA; F, forward primer; R, reverse primer.

## Data Availability

The data used in this study can be provided by the corresponding author upon request.

## Abbreviations

The following abbreviations are used in this manuscript:

NUCB2	Nucleobindin-2
GnRH	Gonadotropin-releasing hormone
KISS1	Kisspeptin
MBH	Mediobasal hypothalamus
POA	Preoptic area
AC/PKA	Adenylate cyclase/protein kinase A
AMPK	AMP-activated protein kinase
PI3K/Akt	Phosphoinositide 3-kinase/protein kinase B
MAPK/ERK1/2	Mitogen-activated protein kinase/extracellular signal-regulated kinase
GT1-7	Mouse Hypothalamic GnRH Neuronal Cell Line
LβT2	Mouse Pituitary Gonadotrope Cell Line
HPG	Hypothalamic–pituitary–gonadal
E_2_	Estradiol
P_4_	Progesterone
T	Testosterone
FSH	Follicle-stimulating hormone
LH	Luteinizing hormone
i.c.v.	Intracerebroventricular
FSHβ	Follicle-stimulating hormone beta-subunit
LHβ	Luteinizing hormone beta-subunit
F-IHC	Fluorescent immunohistochemistry
*UBC*	Ubiquitin C
*18sRNA*	18S ribosomal RNA
SDS-PAGE	Sodium dodecyl sulphate polyacrylamide gel electrophoresis
ELISA	Enzyme-linked immunosorbent assay
